# In Vivo Assembly of a *Dictyostelium* Lamin Mutant Induced by Light, Mechanical Stress, and pH

**DOI:** 10.3390/cells9081834

**Published:** 2020-08-04

**Authors:** Marianne Grafe, Phillip Hofmann, Petros Batsios, Irene Meyer, Ralph Gräf

**Affiliations:** Department of Cell Biology, University of Potsdam, Karl-Liebknecht-Str. 24-25, 14476 Potsdam-Golm, Germany; mgrafe@uni-potsdam.de (M.G.); phillip.hofmann@outlook.com (P.H.); batsios@uni-potsdam.de (P.B.); irene.meyer@uni-potsdam.de (I.M.)

**Keywords:** lamin, NE81, *Dictyostelium*, nuclear envelope, nuclear lamina

## Abstract

We expressed *Dictyostelium* lamin (NE81) lacking both a functional nuclear localization signal and a CAAX-box for C-terminal lipid modification. This lamin mutant assembled into supramolecular, three-dimensional clusters in the cytosol that disassembled at the onset of mitosis and re-assembled in late telophase, thus mimicking the behavior of the endogenous protein. As disassembly is regulated by CDK1-mediated phosphorylation at serine 122, we generated a phosphomimetic S122E mutant called GFP-NE81-S122E-ΔNLSΔCLIM. Surprisingly, during imaging, the fusion protein assembled into cytosolic clusters, similar to the protein lacking the phosphomimetic mutation. Clusters disassembled again in the darkness. Assembly could be induced with blue but not green or near ultraviolet light, and it was independent of the fusion tag. Assembly similarly occurred upon cell flattening. Earlier reports and own observations suggested that both blue light and cell flattening could result in a decrease of intracellular pH. Indeed, keeping the cells at low pH also reversibly induced cluster formation. Our results indicate that lamin assembly can be induced by various stress factors and that these are transduced via intracellular acidification. Although these effects have been shown in a phosphomimetic CDK1 mutant of the *Dictyostelium* lamin, they are likely relevant also for wild-type lamin.

## 1. Introduction

Lamins are type V intermediate filaments and are the major component of the nuclear lamina. They are composed of a short head domain, a long α-helical rod domain (370 amino acids) and a tail domain. Among the intermediate filaments, lamins are characterized by several specific features: a CDK1 consensus sequence preceding the rod domain and, within the tail domain, a nuclear localization sequence (NLS), a globular LTD (lamin tail domain) related to immunoglobulins and a C-terminal CaaX box (= cysteine, two aliphatic aa and X = aa specifying the type of isoprene moiety) [1]. The latter acts as a prenylation signal, which is modified in sequential enzymatic reactions catalyzed by farnesyl transferase, a protease (Ras-converting enzyme 1 or ZMPSTE24) and isoprenyl cysteine methyl transferase (ICMT). In the case of B-type lamins, the mature protein retains its farnesyl or geranylgeranyl residue through its lifetime, while, in the case of A-type lamins, it is removed together with the last 15 amino acids through further cleavage by the ZMPSTE24 protease, in the course of intranuclear filament assembly.

As in cytosolic intermediate filaments, the basic unit for filament assembly is a lamin homodimer which is held together by an α-helical coiled coil formed by the rod domain. Two dimers associate to form tetramers in a head-to-tail orientation, and tetramers assemble into lamin filaments with a thickness of only ~3.5 nm in mouse embryonal fibroblasts [2]. However, in contrast to cytosolic intermediate filaments which invariably assemble into 10-nm filaments with eight tetramers in cross-section, lamins of different organisms exhibit considerable variability in thickness [3]. In animals, filament assembly is coordinated with nuclear import. Here, binding of importin-α to the NLS inhibits too-early filament assembly and ensures that assembly starts only after nuclear import when binding of Ran-GTP triggers the release of importin-α [4]. During interphase, A- and B-type lamins are organized in distinct filament networks which bind to the inner nuclear membrane via various interactions with nuclear envelope transmembrane proteins (NETs) [5]. B-type lamins are additionally attached to the inner nuclear membrane (INM) through their prenyl anchor. During mitosis, lamins are phosphorylated by CDK1, which triggers disassembly of the filament network and allows nuclear envelope breakdown [6]. From an evolutionary point of view, B-type lamins are believed to be more ancient, as the lamin in all organisms possessing only one gene has B-type properties.

We were the first to discover a bona fide lamin in a non-metazoan organism [7]. In a series of experiments including mutational analyses and in vitro assembly studies, we could show that the *Dictyostelium* lamin NE81 is not only evolutionarily related to metazoan lamins and capable of forming filaments of approximately 10 nm in diameter, but also fulfills typical lamin functions [7,8,9,10]. Not surprisingly, NE81 behaved more as a B-type lamin [11]. Using sophisticated bioinformatic tools, soon after our discovery of the first non-metazoan lamin, further lamin-like proteins were identified also in the eukaryotic SAR (Stramenopile, Alveolata, Rhizaria) supergroup [11,12]. This strongly indicates that lamins were already a feature of the last eukaryotic common ancestor (LECA) [13]. 

In the course of our functional analysis of lamin domains, we realized that the deletion of the CaaX-box caused the assembly of three-dimensional protein clusters during interphase. These clusters were either intranuclear, if the NLS was intact [7,14], or cytosolic, if the NLS was inactivated [9,10]. The appearance of NE81 clusters in electron microscopy, the ability of the purified protein to form these clusters in vitro and their behavior during mitosis all indicated that the clusters were not merely aggregates of unfolded protein but real dynamic protein assemblies. During mitosis, all clusters disassembled, regardless of their location. Disassembly required serine 122 within the CDK1 consensus sequence. When point mutated by replacing serine with alanine (non-phosphorylatable mutation), NE81-clusters were present permanently and no longer disassembled during mitosis. FRAP experiments using GFP-NE81 cells revealed that the unmutated INM-associated lamin disassembled in prophase and reassembled in telophase as in animal cells, although *Dictyostelium* cells exhibit a semi-closed mitosis, in which the nuclear envelope becomes permeable for larger proteins in early prophase, when the duplicating centrosome enters a fenestra in the nuclear envelope [8]. GFP-NE81 remained bound to the nuclear envelope. Nevertheless, disassembly of NE81 filaments is required to allow the narrow constriction of the nuclear envelope between the dividing daughter nuclei during karyokinesis. In general, assembly/disassembly of lamins is regulated not only at mitotic onset by CDK1 phosphorylation. There is also experimental evidence for phosphorylation by Cdk5, protein kinase A, Akt, protein kinase C and MAP kinase at various sites within the head and tail domains in various species [6]. Moreover, several studies on mechanotransduction, i.e., how mechanical forces influence the gene expression pattern of cells, have revealed that the phosphorylation-dependent lamin assembly state is responsive to mechanical forces [15,16,17,18,19]. In this work, we show that, in *Dictyostelium*, lamin assembly can be induced not only by mechanical cues, but also by blue light and low pH.

## 2. Materials and Methods

### 2.1. Vector Constructions

The HisMyc-NE81-S122E-ΔNLSΔCLIM vector was previously described in [9]. To generate the corresponding GFP construct, the point mutated NE81 coding sequence was cloned into the N-terminal GFP-fusion vector pIS76 [20]. The red-fluorescent protein mRuby2 [21] was codon-optimized for *Dictyostelium* and cloned into the GFP vector described above using restriction enzymes NheI and SalI, replacing the GFP-cassette. *Dictyostelium* clones containing the plasmid were selected with G418 S (10 µg/mL). Furthermore, successful integration of constructs into the *Dictyostelium* genome was confirmed by Western blot and PCR of genomic DNA. 

### 2.2. Light Microscopy

For fixed samples, 2 × 10^5^ cells were allowed to settle onto 12-mm coverslips for 20 min and fixed with glutaraldehyde as described earlier [22]. All experiments were performed in the dark. Wide-field fluorescence microscopy was performed as described previously [20], using either a Zeiss Axiovert 200 M system equipped with a mercury-halide lamp (Zeiss HXP120), a PlanApo 1.4/100× objective, an Axiocam MRm Rev.3 charge-coupled device (CCD) camera and Axiovision 4.8 software or an Axioobserver System equipped with an LED light source (Zeiss, Colibiri 7), a PlanApo 1.4/100× objective, an Axiocam 506 mono, a piezo stage and ZEN Blue Software (Carl Zeiss Mikroskopie GmbH, Jena, Germany). Deconvolution was performed with the mentioned software packages essentially as described previously [23]. Expansion microscopy (ExM) samples were prepared as described previously [9]. Here, an LCI PlanNeofluar 1.3/63× objective was used. Maximum intensity projections were calculated with Fiji [24]. Live cell imaging was performed with the same system, a Zeiss LSM710 with a C-Apo 1.2/40× objective or a confocal spinning disk system (Cell Observer SD, Carl Zeiss Mikroskopie GmbH, Jena, Germany) equipped with a PlanApo 1.4/100× objective and two Evolve EM-CCD cameras (Photometrics, Tucson, AZ, USA) and 405 (50 mW), 488 (100 mW) and 561 (40 mW) solid state lasers [25]. Live cell samples were prepared according to Samereier (2010) in glass bottom Petri dishes (Fluorodish, WPI, Berlin, Germany) [26]. Approximately 3 × 10^5^ cells per dish were allowed to settle for at least 20 min before the medium was replaced by LoFlo medium pH 6.5 (Formedium, Hunstanton, UK). 

### 2.3. Assembly Experiments

Light induced assembly experiments: Light-induced lamin assembly required high light intensity. As a rule of thumb, for different microscopic systems and light sources, illumination intensity needed to be 4–5 times higher than for normal imaging under gentle conditions (i.e., low bleaching and phototoxicity). For the spinning-disk system, the following protocols were used: assembly occurred by continuous illumination and imaging with 488-nm light at 40% AOTF transmission for 3 min (9 stacks) followed by imaging at 8% AOTF transmission, whereby focus stacks consisting of 15 slices (distance 0.27–0.31 µm) were recorded every 5 min for 13 cycles with 100 ms exposure time per frame. The same strategy was used with the green laser line (561 nm) and near-UV laser line (405 nm), except that the last activation step, i.e., the positive control, was again performed with 40% 488-nm laser for 3 min.

Light induce pH-shift experiments: Live cells were washed twice for 30 min in LoFlo Medium to reduce background fluorescence of HL5c medium. After removal of LoFlo, cells were incubated for 5 min in 20 mM K/K_2_ phosphate buffer (pH 6.1) including 10 µM Carboxy-SNARF-1 AM pH-sensitive dye ([27] Thermo Fisher Scientific, Darmstadt, Germany). The dye solution was exchanged by 20 mM K/K_2_ phosphate buffer (pH 6.1), and cells were viewed on a Zeiss LSM710. Blue light induced assembly of the lamin occurred by 30 s continuous illumination with a HXP100 mercury halide lamp and a 470/40 band pass excitation filter (Zeiss, Jena, Germany). pH-shifts were measured 3 min after blue light stimulation by excitation of Carboxy-SNARF-1 AM at 561 nm by recording a lambda stack from 567 to 606 nm with the spectral detector. Intensity measurements were performed with Fiji [24] whereby the whole fluorescent cell area was considered.

Stress experiments: For oxidative stress experiments, samples were treated as described for light induced assembly except that the LoFlo medium was freshly supplemented with 2 mg/mL sodium ascorbate (final concentration). For osmotic stress experiments, 2 × 10^5^ cells were transferred to a round 12-mm coverslip and allowed to settle for at least 20 min before the medium was replaced either with phosphate buffered saline (hypertonic) or Soerensen buffer (hypotonic) for 1, 4 and 8 h, respectively. Cells were fixed with glutaraldehyde and stained as indicated. 

Cell flattening experiments: Samples were prepared in LoFlo Medium as described for live cell imaging, followed by cell flattening up to one hour by agar overlay as described by Fukui and co-workers [28] in the darkness. The other mechanic stimulus we tested was stress by turbulent shaking culture. Cells in log phase were grown for 24 h in Erlenmeyer baffle flasks on a rotary shaker at 100 rpm in the dark, fixed as described above and stained as indicated. 

pH experiments: Approximately 2 × 10^5^ cells were allowed to settle onto a coverslip for 20 min. Normal HL5c medium (at pH 6.5) was replaced with either HL5c medium pH 5.0 (titrated with HCl) or pH 5.5 (titrated with acetic acid) and incubated for 10, 20 and 30 min. As a control for the reversibility of the protein assembly process, low pH medium was replaced after 30 min with normal HL5c medium pH 6.5, followed by further incubation for 45 min. Samples were fixed with glutaraldehyde and stained as indicated. 

### 2.4. Other Methods

All *Dictyostelium* strains are based on the axenic strain AX2. Cells were cultured in adherent culture in tissue culture flasks and transformed by electroporation as described earlier [9,29].

### 2.5. Antibodies and Conjugates

Monoclonal anti-Myc-tag mouse 9E10 [30] was obtained from ATCC (LGC Standards GmbH, Wesel, Germany). Secondary anti-mouse AlexaFluor 488 was purchased from Thermo Fisher Scientific (Darmstadt, Germany), as well as the DNA dyes Hoechst 33324 and DAPI. 

## 3. Results

**Figure 1:**
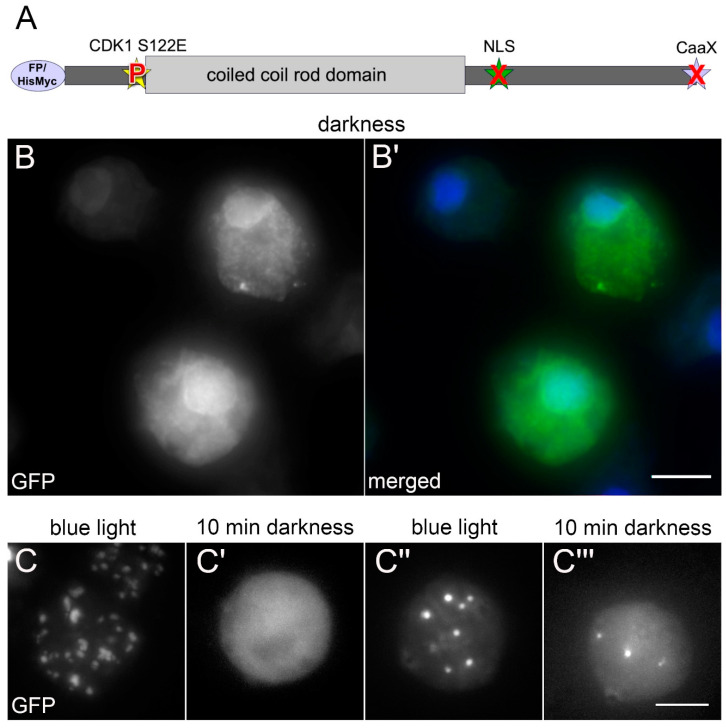
GFP-NE81-S122EΔNLSΔCLIM reversibly forms cytosolic protein assemblies upon blue light illumination. (**A**) Domain overview of the NE81 variant used in this work. FP, fluorescence protein. The phosphomimetic mutation is indicated by a “P” and the disrupted NLS and the deleted CaaX-box are indicated by an “X”. (**B**) Fluorescence microscopy of fixed GFP-NE81-S122EΔNLSΔCLIM cells kept in darkness. Merged images show NE81-S122EΔNLSΔCLIM in green and DAPI staining of DNA in blue. (**C**–**C’’’**) Selected time points of a widefield microscopy live cell imaging series of GFP-NE81-S122EΔNLSΔCLIM cells: (**C**) after 1:30 min excitation with 488 nm; (**C’**) the same cell after 10 min in the dark; (**C’’**) the same cell after 1 min re-excitation with 488 nm; and (**C’’’**) the same cell after 10 min in the dark after re-excitation. Maximum intensity projections of eighteen focal planes at a distance of 0.275 µm are shown. Scale bars = 5 μm.

When we set out to study the phosphorylation dependency of mitotic NE81 disassembly by expression of point mutated GFP or HisMyc-tagged fusion proteins, we initially focused on the canonical CDK1 site (at serine-122 in *Dictyostelium*) known to be required for lamin disassembly in the course of nuclear envelope breakdown in animal cells (Figure 1A). We quickly realized that the results were difficult to interpret if the CaaX-box was functional. In *Dictyostelium*, there is no nuclear envelope breakdown during mitosis, therefore disassembly was not readily visible through the microscope, since the fusion proteins remain at the nuclear envelope also in their disassembled state, most likely by their prenyl anchor. Thus, we expressed NE81 fusion proteins carrying phosphomimetic or non-phosphorylatable point mutations at serine-122 without the CaaX-box (ΔCLIM mutants), with or without the NLS. Here, we made use of the known formation of large, three-dimensional assemblies of the fusion proteins either within the nucleus (with NLS) or in the cytosol (without NLS = ΔNLS mutants), which dissociate during mitosis and reassemble in late telophase [7,10]. The non-phosphorylatable S122A mutation provided the expected results, i.e., the fusion proteins formed clusters which persisted during mitosis [7]. By contrast, the cells expressing the phosphomimetic GFP-NE81-S122E-ΔNLSΔCLIM exhibited an unexpected behavior. Surprisingly, these cells often contained cytosolic clusters of the fusion protein during interphase. During live cell imaging, the tendency of GFP-NE81-S122E-ΔNLSΔCLIM to form cytosolic clusters even increased. This led to the idea that cluster assembly might be elicited by the excitation light, and we investigated this phenomenon systematically.

### 3.1. Assembly of Cytosolic, Phosphomimetic NE81 Is Triggered by Blue Light

When GFP-NE81-S122E-ΔNLSΔCLIM cells were kept in the dark until the start of live cell imaging with 488-nm excitation light, the fusion protein was initially distributed uniformly in the cytosol, with no apparent clustered protein assemblies. Some cells showed an additional GFP signal at the nuclear envelope, which is most likely caused by cytosolic formation of heterodimers of the GFP-fusion protein with endogenous NE81 followed by co-import of the heterodimer into the nucleus (Figure 1B).

However, starting from approximately 30-s exposure to the GFP excitation wavelength, bright GFP foci started to appear and increased in size during continuous illumination (Figure 1C,C’ and Video S1). Sometimes smaller foci coalesced to form larger assemblies. This assembly behavior was similarly observed in GFP-NE81-ΔNLSΔCLIM cells without the phosphomimetic point mutation, where reassembly of the interphase GFP-NE81-ΔNLSΔCLIM protein took place in early telophase and the NE81 clusters increased in size during cytokinesis [10].

**Figure 2:**
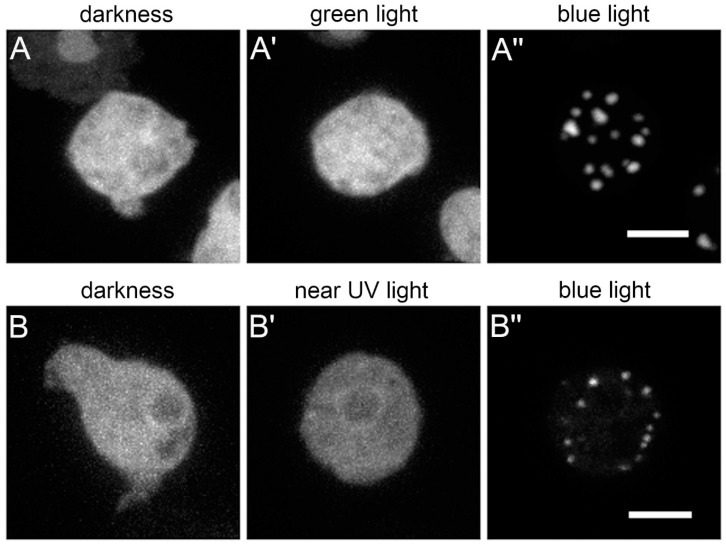
Assembly of GFP-NE81-S122EΔNLSΔCLIM is wavelength-dependent and occurs only upon blue, but not green or near UV light exposure. (**A**,**B**) Selected time points of live cell confocal spinning disk microscopy series comparing response to green light (561 nm) (**A**) or near UV light (405 nm) (B) versus blue light (488 nm). The same representative cell is shown in (**A**–**A’’**) and (**B**–**B’’**), respectively; the left cell shows the situation at t = 0 (recording of one image stack at 8% AOTF transmission). Laser excitations to trigger assembly in (**A’**,**A’’**,**B’**,**B’’**) were for 3 min at 40% AOTF transmission. Maximum intensity projections of fifteen focal planes at a distance of 0.27 µm are shown. Scale bars = 5 µm.

After the excitation light had been turned off for up to 90 min, the bright GFP-fluorescent assemblies disappeared completely, the cells again showed uniform cytosolic fluorescence and had the same appearance as at the beginning of the experiment. After re-exposure to blue excitation light, the formation of GFP-fluorescent clusters started again (Figure 1C’’,C’’’ and Video S2). Next, we wondered whether excitation light induced assembly depended on the wavelength. We tested whether assembly of GFP-NE81-S122E-ΔNLSΔCLIM clusters could be induced with other wavelengths. The two other wavelengths available in our spinning disk system, green light (561 nm) and near ultraviolet light (405 nm), both failed to elicit cluster formation, and the fusion protein remained uniformly distributed in the cytosol (Figure 2; AOTF settings were adjusted accordingly to compensate for differences in laser power of the individual lasers). When the wavelength was again switched to 488 nm, the same cells readily showed cluster assembly as described above (Figure 2). Taken together, cytosolic assembly of GFP-NE81-S122E-ΔNLSΔCLIM clusters was specifically triggered by exposure to blue light (488 nm). This assembly was reversible when cells containing clusters were kept in the dark.

### 3.2. Blue Light-Induced Assembly of Cytosolic, Phosphomimetic NE81 Is Independent of the Fusion Tag

**Figure 3:**
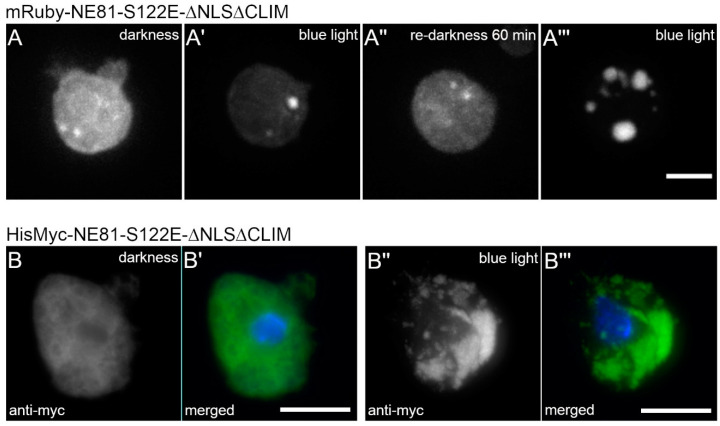
Blue light-induced assembly of NE81-S122EΔNLSΔCLIM is independent of the fusion tag. (**A**–**A’’’**) Selected time points of a spinning disk confocal live cell imaging with mRuby2-NE81-S122EΔNLSΔCLIM cells and excitation at 561 nm. Assembly was triggered by strong (40% AOTF transmission) exposure to 488 nm. (**A**) First recorded image after start of image acquisition; (**A’**) the same cell after 3 min strong (40% AOTF transmission) exposure to 488 nm; (**A’’**) the same cell after 60 min in the dark without 488 nm excitation; and (**A’’’**) the same cell after 3 min re-exposure to strong 488 nm laser light. Scale bar = 5 μm. (**B**–**B’’’**) Expansion microscopy of fixed HisMyc-NE81-S122EΔNLSΔCLIM cells stained with anti-Myc/anti-mouse-AlexaFluor 488 and Hoechst 33342. Merged images (**B’**,**B’’’**) show NE81-S122EΔNLSΔCLIM in green and chromatin in blue; (**B**,**B’**) without light; and, (**B’’**,**B’’’**) after blue light stimulation (band pass filter 450–490 nm). Expansion factors are 3.7 in (**B**) and 3.3 in (**B’**), respectively; scale bar = 5 μm (referring to the original size).

Next, we asked whether the observed effect could be caused by the GFP tag and its spectral properties. We replaced GFP with the mRuby red-fluorescent protein. However, mRuby-NE81-S122E-ΔNLSΔCLIM exhibited the same blue light dependence of protein assembly as the corresponding GFP-fusion protein (Figure 3A), while other light colors including the mRuby excitation wavelength, 561 nm, were ineffective (not shown). 

In the course of our analyses of in vitro lamin assembly, we obtained evidence that, unlike the smaller HisMyc-tag, large tags such as GFP adversely affected the assembly of endogenous NE81 [9]. Thus, we tested whether blue light-induced assembly also occurred in cells expressing HisMyc-NE81-S122E-ΔNLSΔCLIM. Indeed, when treated with the same blue light (488 nm) conditions as the respective GFP strain, these cells exhibited cytosolic HisMyc-NE81-S122E-ΔNLSΔCLIM clusters in a comparable fashion, whereas cells kept in the dark showed a uniform distribution of the tagged protein (Figure 3B). These assemblies were indistinguishable from those observed in HisMyc-NE81-ΔNLSΔCLIM cells without the phosphomimetic point mutation [9].

### 3.3. Mechanical Cell Flattening also Triggers Assembly of Cytosolic, Phosphomimetic NE81

**Figure 4:**
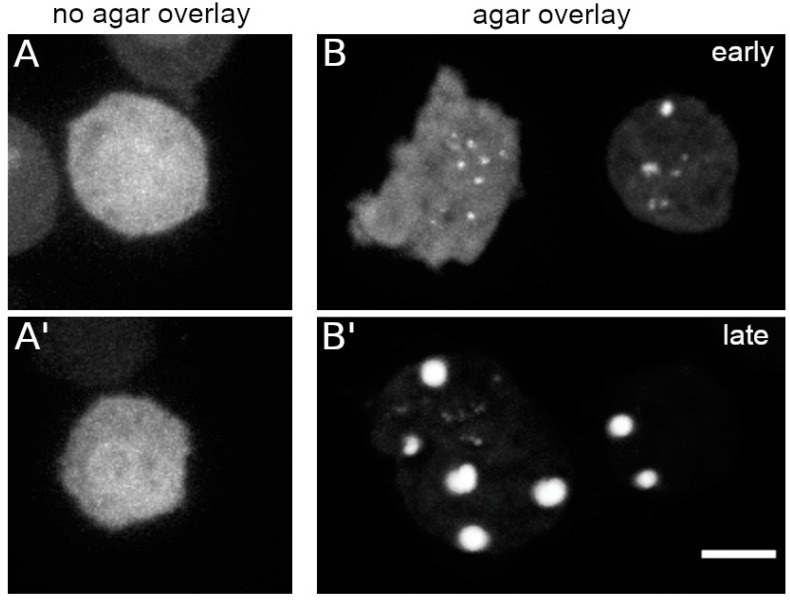
GFP-NE81-S122EΔNLSΔCLIM assembly is triggered by cell flattening. Confocal spinning disk live cell imaging in LoFlo medium: (**A**,**A’**) two control examples prior to agar overlay; and (**B**,**B’**) two examples with two cells each after cell flattening by agar overlay, (**B**) in an early phase up to 1 h and (**B’**) in a late phase (after 1 h). Scale bar = 5 μm.

Live cell imaging of *Dictyostelium* cells is often performed under agar overlay, especially if mitotic events are of interest [26,28]. This prevents the tendency of the mitotic spindle to adopt an oblique position requiring acquisition of large z-stacks. When we viewed GFP-NE81-S122E-ΔNLSΔCLIM cells under agar overlay we observed the formation of cytosolic green-fluorescent assemblies immediately after the start of imaging, even without strong exposure to blue light. The clustering increased over time and was positively correlated with the extent of cell flattening (Figure 4B). Thus, cluster assembly can also be triggered by mechanical cues (Figure 4). However, mechanical stress exerted by shaking culture of cells in Erlenmeyer baffle flasks instead of adherent culture was ineffective in inducing cluster formation in the dark. Reversibility of agar overlay-induced cluster formation could not be tested, since the cells inevitably move away from their position in the glass bottom dishes when the agar sheet is removed.

### 3.4. Assembly of Cytosolic, Phosphomimetic NE81 Is Unaffected by Oxidative or Osmotic Stress

We also asked whether GFP-NE81-S122E-ΔNLSΔCLIM assembly could also be triggered by oxidative or osmotic stress. In principle, it was possible that blue light-induced assembly was caused by oxidative stress, since oxygen radicals are formed under these conditions. However, we observed no difference in assembly behavior in the presence or absence of vitamin C as a potent radical scavenger in live cell imaging. 

This, and the fact that near-UV light, which even increases oxygen radical formation, were ineffective in triggering assembly argued against a role of oxidative stress in this process. Moreover, neither hypotonic stress (Soerensen phosphate buffer) nor hypertonic stress (phosphate-buffered saline) was effective in triggering assembly of HisMyc- or GFP-NE81-S122E-ΔNLSΔCLIM clusters. We concluded that it is not stress in general that affects assembly of this lamin variant.

### 3.5. Assembly of Cytosolic, Phosphomimetic NE81 Is Triggered by Low pH

Thus far, the only known light dependency of cellular processes in *Dictyostelium* occurs in phototactic behavior during development, and this appears to require mitochondrial functions [31]. Since mitochondria could also be involved in pH regulation in addition to the contractile vacuole, we asked whether forced pH decrease could affect GFP-NE81-S122E-ΔNLSΔCLIM assembly as well. This was supported by a brief side note his 1998 paper, where Dr. Inouye mentioned that the exposure of *Dictyostelium* cells to strong excitation light lowers the intracellular pH by an unknown mechanism, but no data were shown [32]. To show that strong blue light exposure causes indeed a decrease in intracellular pH, we incubated GFP-NE81-S122E-ΔNLSΔCLIM cells with the pH-sensitive dye carboxy-SNARF-1 AM [27]. This dye has the advantage that it can be excited with green light and shows an increasing red fluorescence with decreasing pH-values (which avoids overestimation of a pH-shift towards lower values due to artificial bleaching of the dye). However, calibration of SNARF fluorescence intensities with exact pH values are difficult in *Dictyostelium*, since in situ calibrations are error prone due to irreproducibilities when ionophores such as nigericin or monensin are used to allow protons to permeate through the plasma membrane between calibration media and the cytoplasm [32]. 

**Figure 5:**
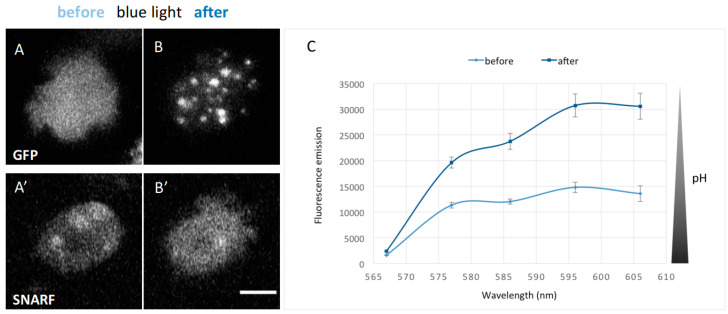
GFP-NE81-S122EΔNLSΔCLIM cells show a decrease in intracellular pH after excitation with blue light and form reversible cytosolic protein clusters. Live cells were loaded with the SNARF pH sensor. Blue light stimulation occurred for 30 s with a mercury halide lamp at a laser scanning confocal microscope (see methods): (**A**,**A’**) cells prior to blue light stimulation; and (**B**,**B’**) after blue light stimulation. The recombinant protein forms assemblies while SNARF fluorescence increases. Scale bar = 5 µm. (**C**) Emission spectra of SNARF-loaded GFP-NE81-S122EΔNLSΔCLIM cells (*n* = 8) excited at 561 nm and measured 3 min after blue light stimulation. The fluorescence emission of SNARF strongly increased between 565 and 610 nm.

**Figure 6:**
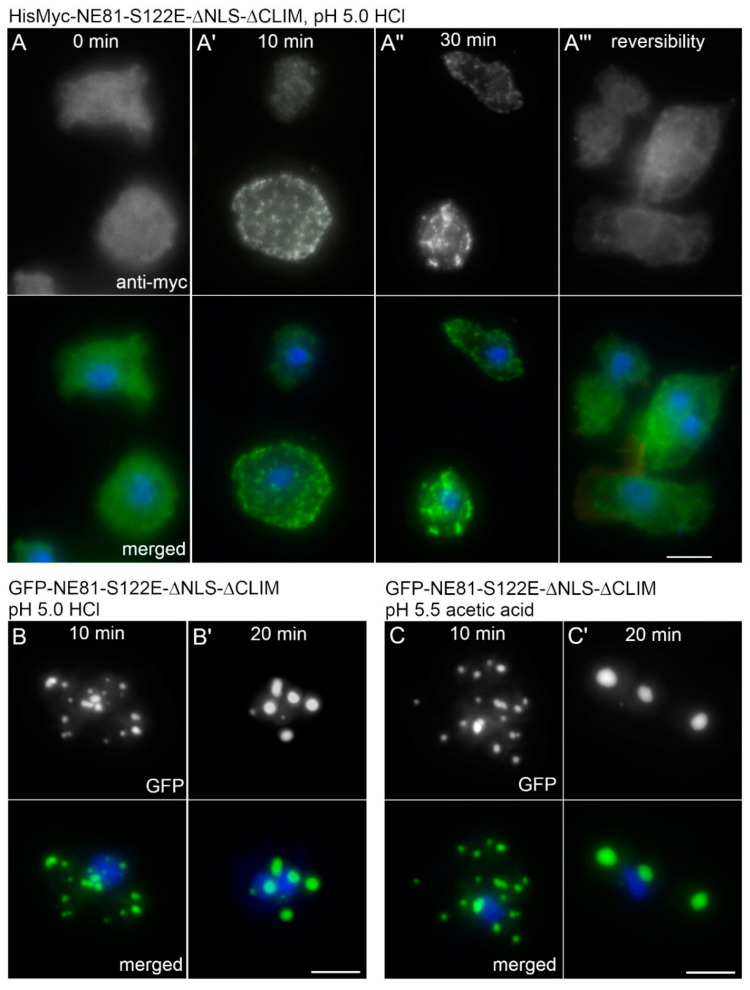
NE81-S122EΔNLSΔCLIM forms reversible cytosolic protein clusters at low pH. (**A**–**A’’’**) Fluorescence microscopy of fixed HisMyc-NE81-S122EΔNLSΔCLIM cells stained with DAPI and anti-Myc/anti-mouse-AlexaFluor 488. Merged images show NE81-S122EΔNLSΔCLIM in green and chromatin in blue: (**A**) control cells in HL5c medium (pH 6.5), t = 0 min; (and **A’**,**A’’**) cells incubated in HL5c medium acidified to pH 5.0 with HCl for 10 min (**A’**) and for 30 min (**A’’**). (**A’’’**) Cells shifted back from pH 5.5 to HL5c medium at pH 6.5 for 45 min to show the reversibility of the process. (**B**,**C**) Fluorescence microscopy of fixed GFP-NE81-S122EΔNLSΔCLIM cells stained with DAPI. Merged images show NE81-S122EΔNLSΔCLIM in green and chromatin in blue. (**B**) Cells were incubated in HL5c medium acidified to pH 5.0 with HCl for the indicated time periods. (**C**) Cells were incubated in HL5c medium acidified to pH 5.5 with acetic acid for the indicated time period. Note that fusion protein clusters increase in size but decrease in numbers in (**B**,**C**), indicating the coalescence of GFP-NE81-S122EΔNLSΔCLIM assemblies with time. For (**B**,**C**), the respective control situation in HL5c medium (pH 6.5) is shown in Figure 1B, Figure 2A,B and Figure 4A,A’. Scale bars = 5 μm.

Despite that, we could show very clearly that intense blue excitation light (450–490 nm) caused both assembly of GFP-NE81-S122E-ΔNLSΔCLIM and a parallel strong reduction in intracellular pH (approximately doubling the SNARF fluorescence in the detection window from 567 to 606 nm; Figure 5). In vegetative cells, intracellular pH values between 7.19 and 7.45 have been reported [32,33]. The pH of standard HL5c medium is 6.5 and the pH of Soerensen phosphate buffer, which is typically used as washing buffer for *Dictyostelium* cells, is 6.1. To analyze the effect of acidification on assembly of GFP-NE81-S122E-ΔNLSΔCLIM or HisMyc-NE81-S122E-ΔNLSΔCLIM, we acidified HL5c medium to pH 5.0 with HCl. In both cell lines, cytosolic cluster formation started after approximately 10 min incubation in the dark in the low pH-medium and increased with time up to 30 min (Figure 6A,B). Cluster formation was fully reversible within 45 min after a medium change back to the standard HL5c medium (Figure 6A). When acetic acid was used for titration instead of HCl to increase the buffering capacity, cluster formation started after 10 min in pH 5.5. After longer incubation times (20 min), fewer but larger clusters were visible (Figure 6B,C), which reflects the known tendency of GFP-NE81-S122E-ΔNLSΔCLIM clusters to coalesce. There was no obvious difference in behavior between the GFP-tagged and HisMyc-tagged versions of the protein.

Taken together, cytosolic assembly of tagged NE81-S122E-ΔNLSΔCLIM could be induced by incubation of cells in low-pH medium to the same extent as by exposure to blue light and exhibited a comparable reversibility. 

## 4. Discussion

For the first time, we provide evidence that assembly of lamins in vivo can principally be induced by low pH and stress factors such as illumination with visible blue light or mechanical flattening of cells by agar overlay. Thus far, a role of pH in lamin assembly has only been shown in vitro, when lamin solubility was increased at high pH. This behavior has been reported for example for *C. elegans* lamin [34] and for *Dictyostelium* HisMyc-NE81-ΔNLSΔCLIM [9]. There are also several reports on mechanotransduction describing a response of the lamin assembly state to mechanical forces [18,19]. Although it became clear that these responses involve changes in the lamin phosphorylation state, many aspects in this mechanotransduction pathway remain elusive. Based on our results, we now hypothesize that, for all environmental cues known to influence lamin assembly, the intracellular pH could be the actual effector. 

In in vitro experiments, *Dictyostelium* lamin assembly was affected only by salt concentration, concentration of the lamin protein and pH [9], while blue light had no effect (not shown). In vivo, of the mentioned parameters, only the pH is likely to quickly respond to the environmental stress factors we tested, i.e., illumination with blue light and mechanical flattening of cells. 

How could blue light exposure cause a decrease of intracellular pH in *Dictyostelium* amoebae? The phenomenon was already mentioned in 1998 [32] and we could show that strong blue excitation light (in the range of 450–490 nm) caused a rapid decrease of the intracellular pH. However, the underlying mechanism remains elusive. No light-dependent process is known in the amoeboid state of *Dictyostelium*; however, during development, the slug state clearly exhibits positive phototaxis. Thus, this organism is principally capable of sensing light, but how it achieves this is still not clear. One line of evidence leads to mitochondria. Already in 1978, Manabe and Poff described blue light sensitivity (photoreduction) of *Dictyostelium* cytochrome b in in vitro experiments [35]. Cytochrome b is part of the proton-translocating respiratory chain complex III of the inner mitochondrial membrane. Furthermore, in 2011, Francione and Fisher observed defective phototaxis in slugs of *Dictyostelium* mutants carrying a knock-down of cytochrome b [31]. Although the contractile vacuole is considered the major pH regulator of *Dictyostelium* amoebae, mitochondria could also contribute to intracellular pH regulation. Mitochondria are involved in intracellular acidification during apoptosis, after various stimuli [36]. Moreover, treatment of *Dictyostelium* cells with the mitochondrial uncoupler and protonophore carbonyl cyanide m-chlorophenylhydrazone (CCCP) caused significant acidification of the cytoplasm [33,37]. Thus, strong blue light may cause (partial) uncoupling of proton transport in the respiratory chain by cytochrome b and resulting decrease in intracellular pH. Since near-UV light (405 nm) failed to induce the assembly of tagged NE81-ΔNLSΔCLIM, and the addition of antioxidants had no effect, it is unlikely that blue light illumination causes mitochondrial damage through phototoxicity. The role of mitochondria and cytochrome b certainly deserves further investigation, which, however, will be complicated by the fact that cytochrome b is encoded by the mitochondrial genome, resulting in instability of knock-down mutants. 

How may cell flattening be related to a decrease in intracellular pH? The agar overlay effect could be due to inhibition of contractile vacuole function. It is a well-known phenomenon that cells viewed under agar overlay have a tendency to grow in volume and burst. As the contractile vacuole serves as volume regulator of *Dictyostelium* cells, this indicates that the function of this organelle is disturbed under agar overlay. The contractile vacuole is also responsible for cellular pH regulation and osmoregulation. Agar overlay could cause cytosolic acidification when cells are kept in media at a pH below the normal cytosolic pH, which is usually the case in *Dictyostelium* standard media (Soerensen buffer, pH 6.1, LoFlo medium pH 6.5). Mechanosensitivity of the contractile vacuole might employ mechanosensitive calcium channels in the vacuolar membrane. The involvement of mechanosensitive calcium channels in the oscillatory behavior of the contractile vacuole was discussed by Plattner (2013) [38]. It is possible that contractile vacuole function is inhibited by continuous activation of mechanosensitive calcium channels that would normally open only upon increased membrane tension during the filling process of the vacuole, to initiate its discharge. 

Flattening of cells and nuclei by agar overlay was recently recognized also in mammalian cells as a specific cue to promote cell growth through nuclear mechanotransduction [39]. In this work, Aureille and co-workers showed that nuclear flattening induced transcription factors such as TEAD and AP1, which in turn induced target genes promoting cell cycle progression. A role of alterations in intracellular pH in the signal transduction process, which would be interesting with respect to our new results, was not investigated. Thus, whether contractile vacuole inhibition by cell flattening in *Dictyostelium* and transcriptional changes by mechanotransduction after nuclear flattening in mammalian cells are related remains unclear. However, both observations are in line with the observation that shearing forces exerted by shaking culture of *Dictyostelium* cells in Erlenmeyer baffle flasks failed to induce cluster formation of tagged NE81-S122E-ΔNLSΔCLIM.

## 5. Conclusions

Taken together, our results show that lamin assembly can be induced by exposure to blue light, cell flattening and acidification of the medium. All these conditions likely converge at a direct or indirect decrease in intracellular pH, and it is the latter which directly favors assembly of lamin filaments. This is also supported by the fact that, of all cues tested in this work, pH is the only one affecting lamin assembly also in vitro, i.e., in a cell-free environment. These effects are difficult to demonstrate in wildtype cells, since the majority of wildtype lamin protein is in the assembled state during interphase. Nevertheless, we believe that, although these effects are shown in a phosphomimetic lamin mutant, regulation of assembly by pH changes is likely to be relevant also for wildtype lamin. 

## Supplementary Materials

The following are available online at https://www.mdpi.com/2073-4409/9/8/1834/s1, Video S1: GFP-NE81-S122EΔNLSΔCLIM forms cytosolic protein assemblies upon blue light illumination. Confocal spinning disk live cell imaging of GFP-NE81-S122EΔNLSΔCLIM cells exposed to blue light at time point 0 s. Maximum intensity projection calculated from nine focal planes (z-distance 0.27 µm). Video S2: Reactivation of GFP-NE81-S122EΔNLSΔCLIM assembly after a dark period. The same cell as in Video S1 is shown. Imaging conditions were as described for Video S1.
