# Supplementary figures and images for: In Vivo Assembly of a Dictyostelium Lamin Mutant Induced by Light, Mechanical Stress, and pH
Source: Cells. 2020 Aug 4;9(8):1834. doi: 10.3390/cells9081834 (PMC7464662; doi:10.3390/cells9081834)

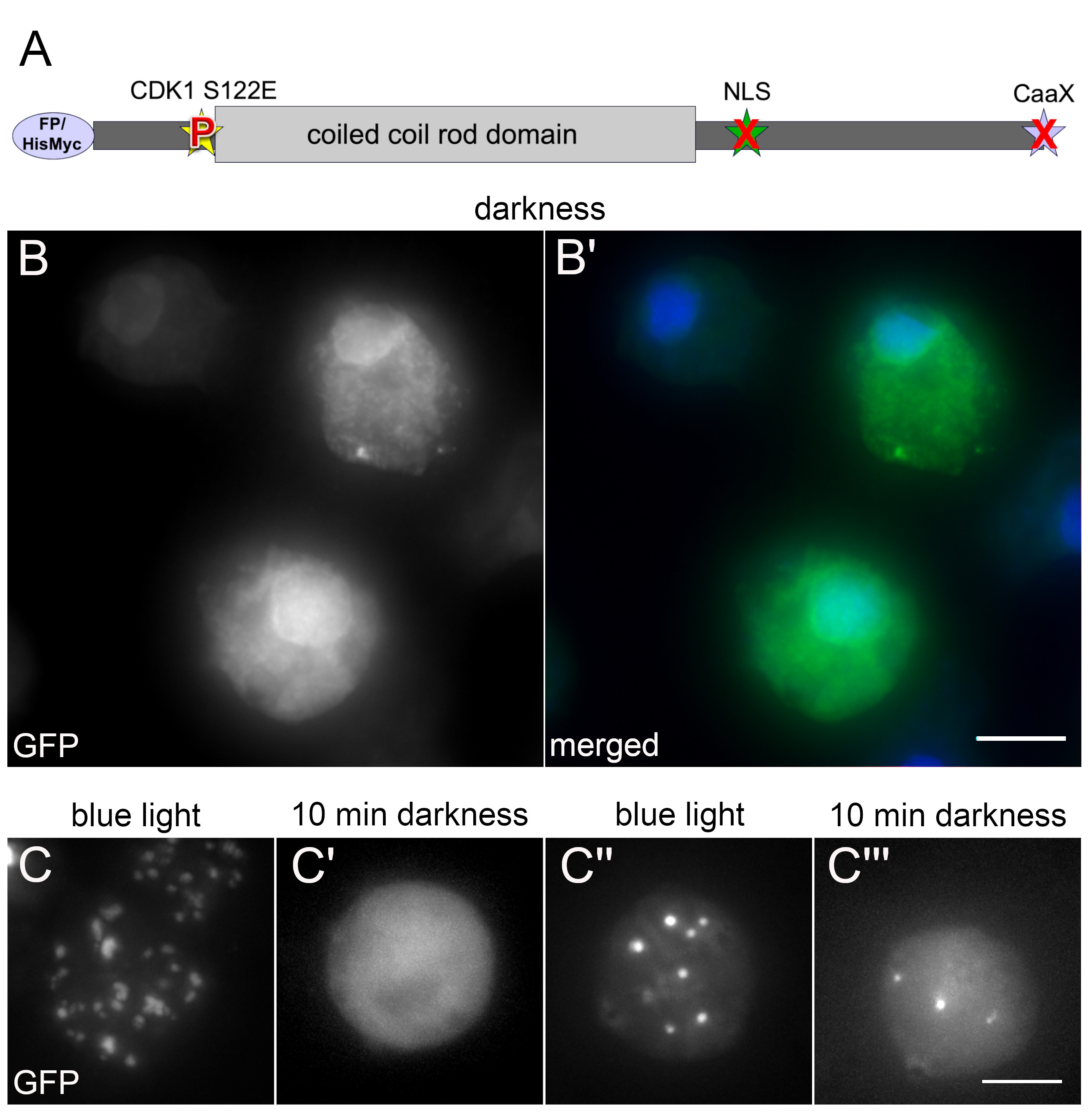

Supplement: Supplementary file 1 [file cells-09-01834-s001.zip › Fig1oR.jpg]

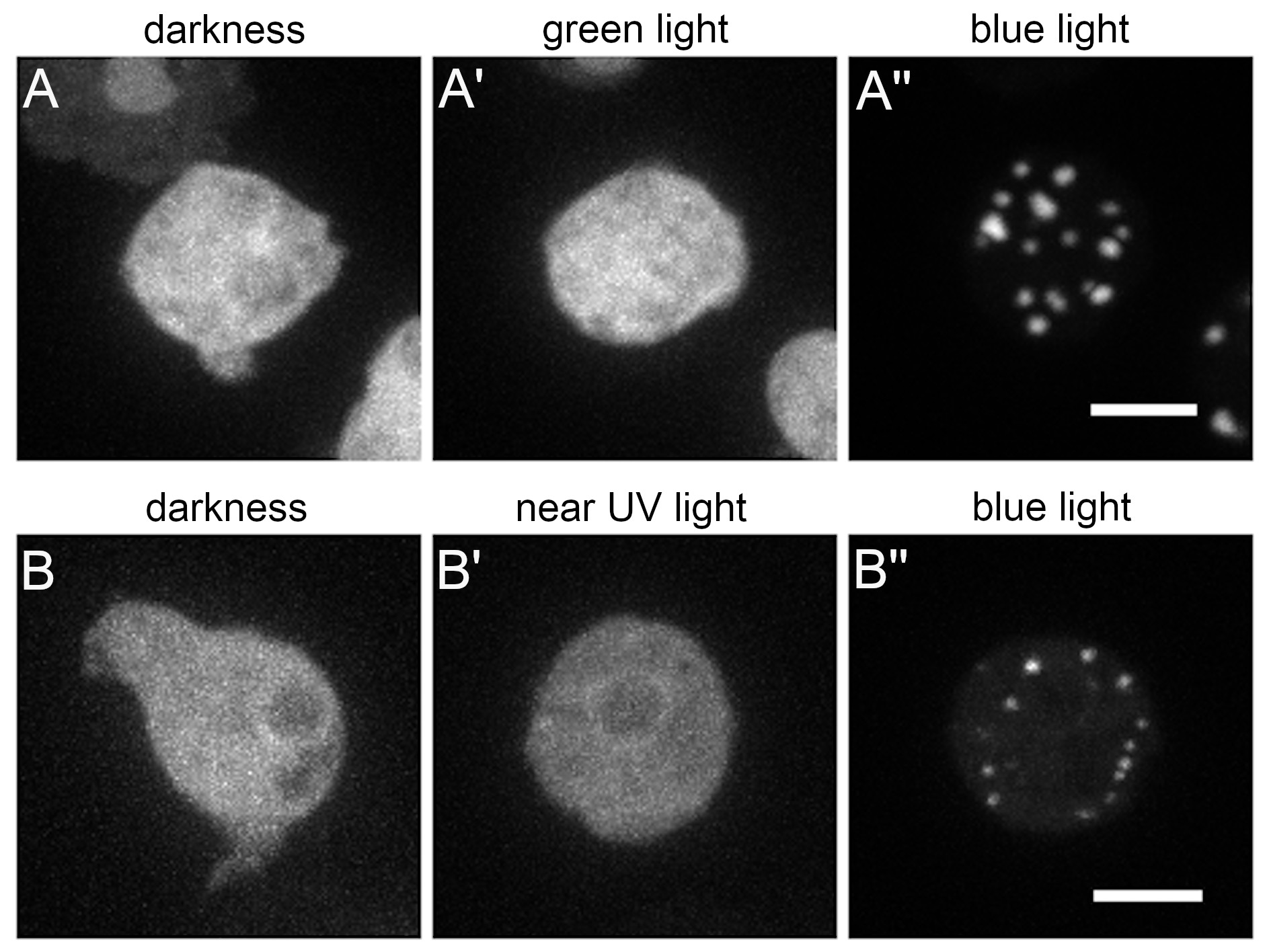

Supplement: Supplementary file 1 [file cells-09-01834-s001.zip › Fig2.jpg]

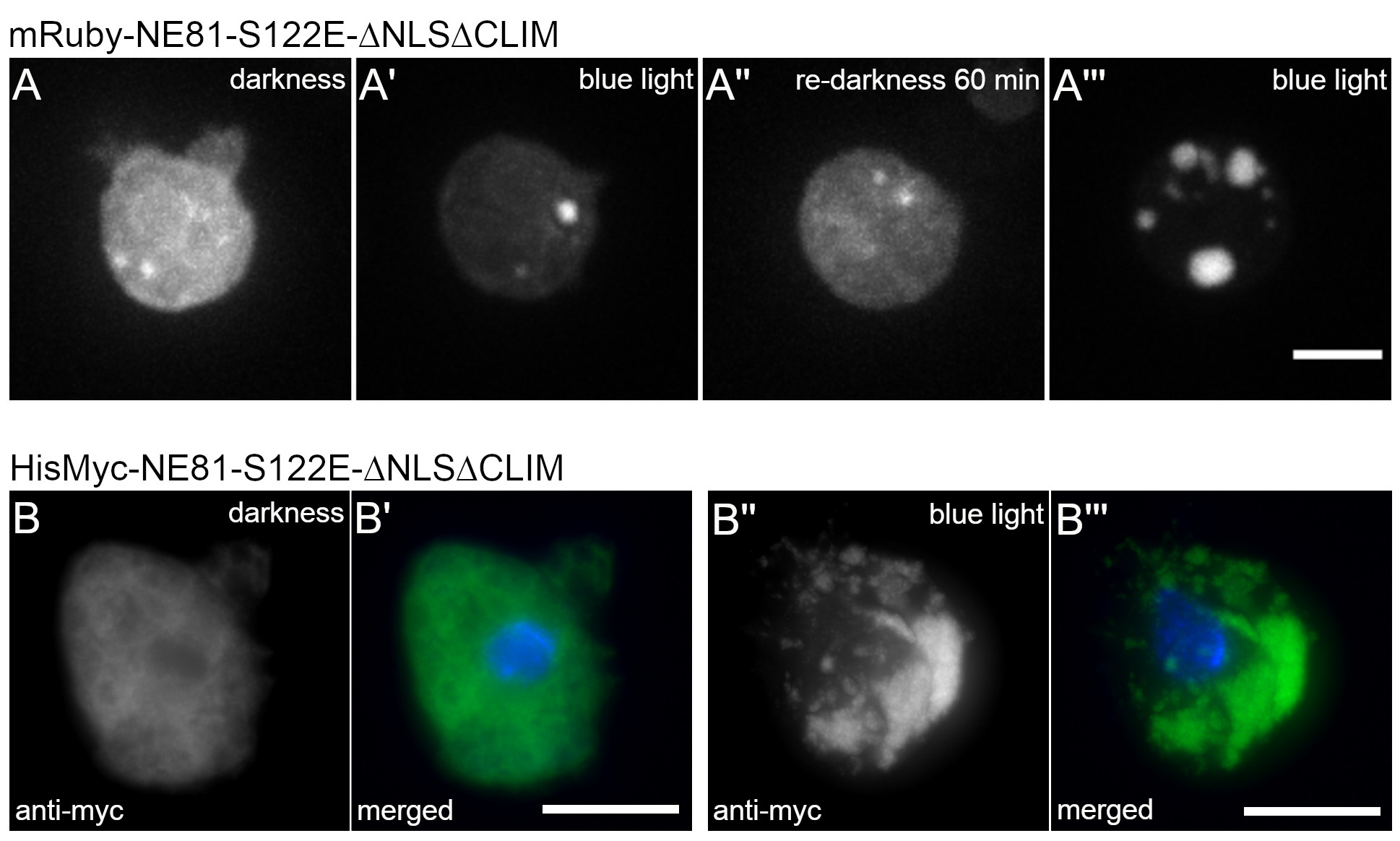

Supplement: Supplementary file 1 [file cells-09-01834-s001.zip › Fig3oR.jpg]

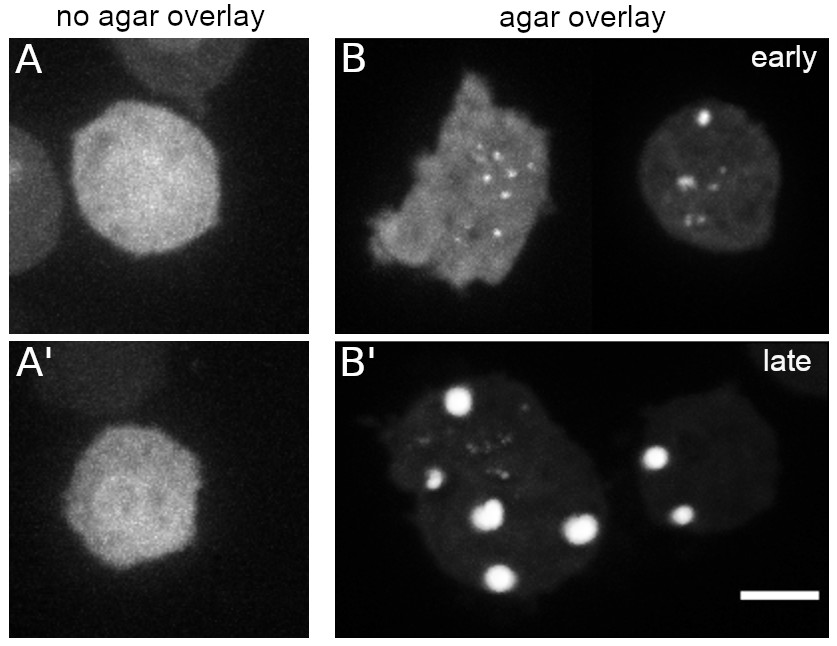

Supplement: Supplementary file 1 [file cells-09-01834-s001.zip › Fig4AB.jpg]

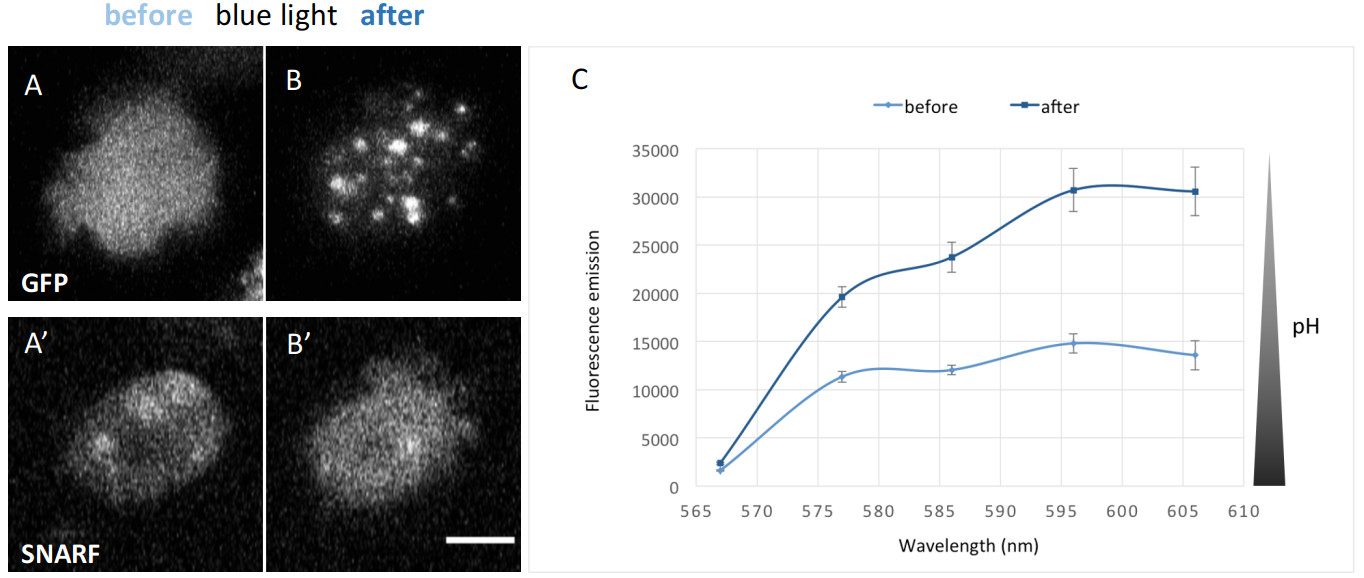

Supplement: Supplementary file 1 [file cells-09-01834-s001.zip › Fig5.jpg]

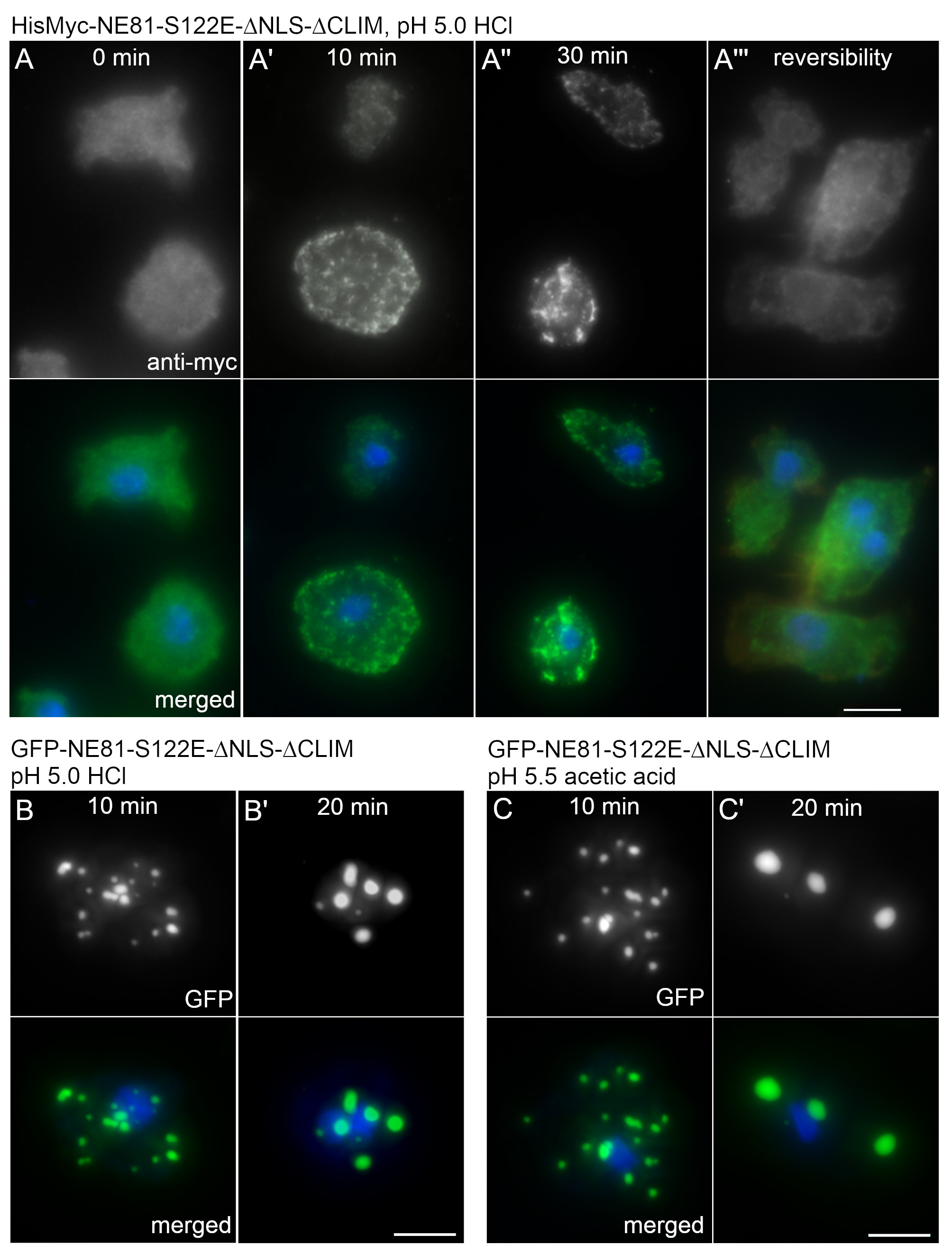

Supplement: Supplementary file 1 [file cells-09-01834-s001.zip › Fig6.jpg]
